# Genetic polymorphisms of innate immunity-related inflammatory pathways and their association with factors related to type 2 diabetes

**DOI:** 10.1186/1471-2350-12-95

**Published:** 2011-07-14

**Authors:** Paul Arora, Bibiana Garcia-Bailo, Zari Dastani, Darren Brenner, Andre Villegas, Suneil Malik, Timothy D Spector, Brent Richards, Ahmed El-Sohemy, Mohamed Karmali, Alaa Badawi

**Affiliations:** 1 Office for Biotechnology, Genomics and Population Health, Public Health Agency of Canada, 180 Queen Street West, Toronto, M5V 3L7, Canada; 2 Dalla Lana School of Public Health, University of Toronto, College Street, Toronto, M5T 3M7, Canada; 3 Department of Nutritional Sciences, University of Toronto, College Street, Toronto, M5S 3E2, Canada; 4 Department of Epidemiology, Biostatistics and Occupational Health, McGill University, Pine Avenue West, Montreal, H3A 1A2, Canada; 5 Department of Twin Research and Genetic Epidemiology, King's College London, St. Thomas's Hospital, Westminster Bridge Road, London, SE1 7EH, UK; 6 Department of Medicine, McGill University, Pine Avenue West, Montreal, H3A 1A1, Canada

## Abstract

**Background:**

Type 2 diabetes mellitus (T2DM) has been linked to a state of pre-clinical chronic inflammation resulting from abnormalities in the innate immune pathway. Serum levels of pro-inflammatory cytokines and acute-phase proteins, collectively known as 'inflammatory network', are elevated in the pre-, or early, stages of T2DM and increase with disease progression. Genetic variation can affect the innate immune response to certain environmental factors, and may, therefore, determine an individual's lifetime risk of disease.

**Methods:**

We conducted a cross-sectional study in 6,720 subjects from the TwinsUK Registry to evaluate the association between 18 single nucleotide polymorphisms (SNPs) in five genes (*TLR4*, *IL1A*, *IL6*, *TNFA*, and *CRP*) along the innate immunity-related inflammatory pathway and biomarkers of predisposition to T2DM [fasting insulin and glucose, HDL- and LDL- cholesterols, triglycerides (TGs), amyloid-A, sensitive C-reactive protein (sCRP) and vitamin D binding protein (VDBP) and body mass index (BMI)].

**Results:**

Of 18 the SNPs examined for their association with nine metabolic phenotypes of interest, six were significantly associated with five metabolic phenotypes (Bonferroni correction, *P ≤ 0.0027*). Fasting insulin was associated with SNPs in *IL6 *and *TNFA*, serum HDL-C with variants of *TNFA *and *CRP *and serum sCRP level with SNPs in *CRP*. Cross-correlation analysis among the different metabolic factors related to risk of T2DM showed several significant associations. For example, BMI was directly correlated with glucose (r = 0.11), insulin (r = 0.15), sCRP (r = 0.23), LDL-C (r = 0.067) and TGs (r = 0.18) but inversely with HDL-C (r = -0.14). sCRP was also positively correlated (*P < 0.0001*) with insulin (r = 0.17), amyloid-A (r = 0.39), TGs (r = 0.26), and VDBP (r = 0.36) but inversely with HDL-C (r = -0.12).

**Conclusion:**

Genetic variants in the innate immunity pathway and its related inflammatory cascade is associated with some metabolic risk factors for T2DM; an observation that may provide a rationale for further studying their role as biomarkers for disease early risk prediction.

## Background

Type 2 diabetes mellitus (T2DM) represents a significant global health problem. It is estimated that six people die every minute from the disease worldwide, a figure that will soon make the disease one of the world's most prevalent causes of preventable mortality [1]. The incidence of T2DM increases with age, obesity, physical inactivity and unhealthy diet, and is elevated among certain ethnocultural groups (Hispanics, Africans, and Aboriginals). The disease rates are also currently increasing among children [2,3]. T2DM is primarily caused by impaired glucose tolerance (IGT), which leads to islet β-cell dysfunction and their subsequent destruction. The ensuing insulin deficiency as a result of β-cell dysfunction impacts skeletal muscle, liver and adipose tissues [4]. Among individuals with IGT, both host (primarily genetic) and environmental factors contribute to the progression of this condition to insulin resistance to T2DM [5-9].

Within the last decade, a hypothesis was proposed to explain the pathogenesis of T2DM by connecting the disease to a state of preclinical chronic inflammation that results from abnormalities in innate immunity pathways [10,11]. Activation of innate immunity promotes various inflammatory reactions that provide the body's first line of defense against microbial, chemical and physical injury, leading to damage repair, isolation of microbial threats and restoration of tissue homeostasis [12,13]. The systemic reaction of innate immunity, known as the 'acute-phase response', is initiated when exogenous threats, such as pathogens or certain dietary factors, are detected by pattern-recognition receptors such as toll-like receptors (*e.g*., TLR-4). The binding of the exogenous molecules by TLR-4 triggers the release of pro-inflammatory cytokines, like tumour necrosis factor (TNF)-α, interleukin (IL)-1β and IL-6 [14]. These cytokines are derived primarily from activated macrophages and can directly enhance insulin resistance in adipocytes, muscle and liver cells [15,16]. Activation of macrophages towards an inflammatory phenotype results in further cytokine synthesis and release [17,18]. Cytokines down-regulate major anabolic cascades involved in insulin signalling and can, subsequently, mediate adipocyte insulin resistance [17,18]. Ultimately, this process contributes to a systemic disruption of insulin homeostasis, leading to a status of impaired glucose tolerance [19]. Cytokines also trigger the synthesis of acute-phase inflammatory proteins such as C-reactive protein (CRP) and serum amyloid-A. Pro-inflammatory cytokines and acute-phase reactants, collectively known as "inflammatory network", play a role in initiating the early stages of T2DM and are known to increase with disease progression.

Genetic variation in the innate immunity pathway may affect the extent of its activation and response upon exposure to stimuli and, consequently, influencing the lifetime risk of a given disease [20]. For example, genetic variations along the innate immunity pathway may lead to an altered response to dietary factors (*e.g.*, fatty acids) known to bind to *TLR4 *and initiate a downstream signaling cascade leading to the synthesis of pro-inflammatory cytokines [21]. As mentioned above, cytokines contribute to initiating a state of impaired glucose tolerance, insulin resistance and a subsequent elevated risk of T2DM. Elucidating the relationship between genetic variation in innate immunity, inflammation, and biochemical indices of T2DM is, therefore, critical to evaluate the feasibility of employing innate immunity-related genetic polymorphisms and inflammatory factors in predicting T2DM risk. Such knowledge might also provide opportunities for the development of novel approaches for disease prevention in the general population and vulnerable sub-populations, e.g., by using nutritional and/or therapeutic factors that attenuate low-grade chronic inflammation [21].

The present study was undertaken in an attempt to explore the association between polymorphisms in various genes along the innate immunity-related inflammation cascade and the metabolic phenotypes associated with risk of T2DM in order to evaluate their possible utility as biomarkers in disease early risk prediction.

## Methods

We conducted a cross-sectional analysis of the association between factors that can be linked to early risk of T2DM (as defined by the profile of risk-related metabolic phenotypes) and genetic variation in candidate genes along the innate immunity-related inflammatory pathway, using data from the TwinsUK Registry [22].

### Study subjects

The UK Adult Twin Registry (TwinsUK) is a database of over 10,000 monozygotic and dizygotic twins aged 13 to 83 years, with a mean age of 48 years [22]. Initiated as an investigation of osteoporosis and osteoarthritis, the TwinsUK cohort evolved to include investigation of the genetics of the metabolic syndrome and cardiovascular disease as one of its main research goals [23]. The cohort consists mostly of Caucasian female volunteers from throughout the United Kingdom and Ireland who were recruited via media campaigns [22]. This research conformed to the ethical standards of the Helsinki Declaration. The study protocol was approved by St. Thomas' Hospital Local Research Ethics Committee and all participants gave written informed consent prior to commencing the study. Recruitment and data collection began in 1993, and nearly half of the cohort has been clinically examined on at least two occasions [22]. The specific data for this study were available through a collaboration initiated by the Public Health Agency of Canada (A.B.) with McGill University (B.R.) and King's College London (T.D.S.). As such, the investigators had restricted access to the selected genotypic and phenotypic data that were analyzed in this study. We only included cohort members aged sixteen years and older. The population has been found to be representative of singletons in the UK population with respect to prevalence of chronic disease and lifestyle characteristics [24].

### Phenotypic and genotypic data

The serum biochemical indices and phenotypes of predisposition to T2DM [fasting insulin and glucose, HDL-cholesterol and LDL-C, triglycerides (TGs), the acute phase proteins amyloid-A, highly sensitive C-reactive protein (sCRP) and vitamin D binding protein (VDBP) in addition to body mass index (BMI)] were obtained from an extensive set of phenotypes available for the TwinsUK study http://www.twinsUK.ac.uk. We did not have an access to variables related to subject prescription drug use and we, therefore, were unable to consider this factor in our analysis. Furthermore, study subjects with evidence of diabetes (fasting glucose measurement of > = 7 mmol/L) were excluded from the analysis (oral glucose tolerance test data were not available) and this group is likely to include a substantial fraction of those on lipid- or blood pressure-lowering medications from the cohort. Furthermore, genome-wide genotyping was performed in the population using the Illumina Hap317K chip [25]. A selected set of SNPs (or their proxies) was chosen from among the genotypes available through this genome-wide association scan (GWAS).

### SNP identification

Eighteen SNPs were identified in five innate immunity candidate genes (*TLR4, IL1B*, *IL6*, *TNFA*, and *CRP*). The studied SNPs were selected based upon their previously published effects on increasing the serum levels of a range of inflammatory markers and their potential role in the aetiology of T2DM [22,26-35]. We carried out an association analysis on nine preselected metabolic markers with the following 18 SNPs [proxy]: *IL1A *(rs17561, rs1609682 [rs10496444], rs2856838 [rs6746923], rs1878321 [rs4848300]); *IL-6 *(rs1554606, rs2069837, rs1474347 [rs7801406], rs2069827 [rs11766273]); *TLR4 *(rs1927906, rs1927914, rs4986790, rs1554973; TNFα (rs3093662, rs1800630 [rs2259435]); and *CRP *(rs2808630, rs1205 [rs2794520], rs1417938 [rs12093699], rs3093059 [rs11265260]). Where the SNPs of interest were not available on the Hap317K chip, we identified proxy SNPs in strong linkage disequilibrium (LD) with those of interest (>80%). LD was assessed by spectral decomposition of pair-wise matrices (Additional file 1 Figure S1a - 1e), so that the 18 SNPs examined here represented the variation of the above-identified 27 candidate loci within the five studied genes [36,37].

### Statistical methods

All statistical analyses were performed using SAS software (SAS Institute Inc., Cary, NC, USA.). The normality of the variables was assessed, and all phenotypes, except HDL-C and LDL-C, were log_e_-transformed. Since a large majority of our sample (>90%) consisted of females, we did not stratify any of the analyses by sex. Covariates considered for the multivariable linear regression analyses were age, sex, and BMI. If these covariates were associated with the outcome phenotype in a univariate analysis, they were included in a subsequent multivariable analysis of association between SNP(s) and phenotype(s). VDBP level was adjusted for age and LDL-C, whereas TGs were adjusted for age and BMI. The remainder of the covariates were all adjusted for age, sex, and BMI. Where we observed multiple significant associations between SNPs in a single gene and a given outcome, we further investigated both the independent effects of each SNP as well as the potential joint effects of SNP combinations within the same gene simultaneously. To test for independent effects, we considered multiple SNPs into the regression models to observe whether significance remained. We created multiplicative interaction terms to test for significant joint effects in two-way interactions between genotypes. As our dataset included twin pairs, we accounted for non-independence of observations using generalized estimating equations with a compound symmetry covariance structure similar to a previously published study of a twins cohort [38]. Spearman correlations were calculated among all the adjusted metabolic indices of T2DM examined in the present study. All statistical tests assumed an additive effect of the allele using maximum-likelihood linear regression analysis. In total, nine independent statistical models were evaluated. Within each model, 18 independent statistical tests were performed at the selected loci. Thus, statistical significance was declared at *P ≤ 0.0027 *by applying a Bonferroni correction (cutoff p-value = 0.05/18). The Bonferroni correction is very conservative and results in highly reduced statistical power to detect differentiation among pairs of sample collections [38]. The Benjamini and Yekutieli (B-Y) false discovery rate (FDR) method is similar to the Bonferroni correction, but it is a less conservative statistical adjustment for multiple comparisons and was, therefore, applied in the present study in addition to the Bonferroni test (B-Y cutoff p-value = 0.014) [39]. The B-Y critical value (B-Y_crit_) is its own modified FDR measure and is calculated as B-Y_crit _= α/∑(1/i). Where "α" = 0.05 and "I" varies from 1 to 18 representing the number of tests conducted in a given regression model. For alpha = 0.05 and i = 18, B-Y_crit _= 0.014. Mean values for the examined phenotypic measures, stratified by genotype for each of the 18 selected SNPs, as well as minor allele frequencies (MAF), are presented in Additional file 2, Table S1.

## Results

The clinical characteristics of the study subjects (*n *= 6,720) are summarized in Table 1. The age of the study subjects ranged between 16 and 83 years. The majority of the subjects were female (90.3%). The overall BMI for the cohort was 25.2 kg/m^2^, with 53.7% of the subjects showing normal BMI measures between 18.5 and 24.9 kg/m^2^. All serum biochemical indices of predisposition to T2DM were, on average, within the normal clinical range.

**Table 1:** Characteristics of the study subjects from the TwinsUK cohort

Measure	*n*	Mean ± SE^2^	Range
Age (years)	6,720	46.2 ± 0.16	16.1 - 83.8
Sex			
Male	653	9.7%	
Female	6,067	90.3%	
Fasting glucose (mmol/L)	6,623	4.6 ± 0.01	2.1 - 6.7
Triglycrides (mmol/L)	5,541	1.1 ± 0.01	0.15 - 8.9
Fasting Insulin (μIU/ml)	2,414	11.9 ± 0.31	2 - 177
LDL-C (mmol/L)	1,780	3.3 ± 0.02	0.3 - 6.9
HDL-C (mmol/L)	4,878	1.5 ± 0.01	0.2 - 3.1
sCRP (mg/L)	5,464	3.1 ± 0.09	0.0 - 161
Amyloid-A (mg/L)	360	6.7 ± 0.41	2 - 91
VDBP (ng/L)	1,730	301 ± 1.23	190 - 602
BMI (Kg/m^2^)	4,710	25.2 ± 0.07	13.2 - 52.4

^1^Study population excluded subjects with fasting glucose of ≥ 7 mmol/L (i.e., possible diabetics) and those under 16 years of age.

^2^Values represent unadjusted mean ± standard error of *n *subjects.

Significant associations between genotypes of the selected SNPs and metabolic phenotypes are presented in Table 2. Of the 18 SNPs examined against each of the nine metabolic indices of T2DM, only six demonstrated a significant association with one or more phenotype after B-Y correction (p < 0.014). The specific associations were between SNPs in *IL6*, *TNFA *and *CRP *and fasting insulin, TGs, HDL-C and sCRP. After Bonferroni adjustment (*p ≤ 0.0027*), the only remaining associations were between the SNPs in *CRP *and sCRP. Each additional allele of the *CRP *rs1205 was associated with 39.2% (95%CI: 29.5-47.6%) decrease in serum CRP level while each additional allele of *CRP *rs1417938 was associated with 67.3% (95%CI: 44.5-93.8%) increase in serum CRP level. The independent effects of these two SNPs on serum CRP level remained significant after adjustment for each other (p_rs1205 _= 0.0002; p_rs1417938 _= <0.0001).

**Table 2:** Association between a selected set of SNPs along the innate immunity-related inflammatory pathway and a number of metabolic phenotypes related to risk of type II diabetes mellitus.

		Metabolic Markers^1^									
		**Insulin^2,3^**		**Triglycerides^2,4^**		**HDL-C^4^**		**sCRP^2,3^**		**VDBP^2^**	

**Gene**	**SNP (Proxy)**	**β (*p*)**	**Model *n***	**β (*p*)**	**Model *n***	**β (*p*)**	**Model *n***	**β (*p*)**	**Model *n***	**β (*p*)**	**Model *n***

*IL6*	rs1554606			0.0574 (0.0103)	3,039						
	rs1474347 (rs7801406)	-0.155 (0.0078)	2,489								
*TNFA*	rs3093662	0.316 (0.0034)	1,583								
	rs1800630 (rs2259435)					-0.0883 (0.0068)	1,945				
*CRP*	rs1205 (rs2794520)							-0.498 (<0.0001)^5^	1,959		
	rs1417938 (rs12093699)					0.0699 (0.0079)	1,946	0.515 (<0.0001)^5^	1,951	0.0383 (0.0134)	1,082

**^1^**Values represent regression coefficient (β) and p-values in parentheses. Metabolic phenotypes and SNPs with no significant association were not presented. Bonferroni correction level of statistical significance is p < 0.0027; B-Y correction level of statistical significance is p < 0.014.

**^2^**Natural logarithm of measures.

**^3^**Adjusted for BMI, age and sex.

**^4^**Adjusted for BMI and sex.

**^5^**Associations remained significant (p < 0.05) after adjustment for other SNPs in the gene.

**Figure 1:**
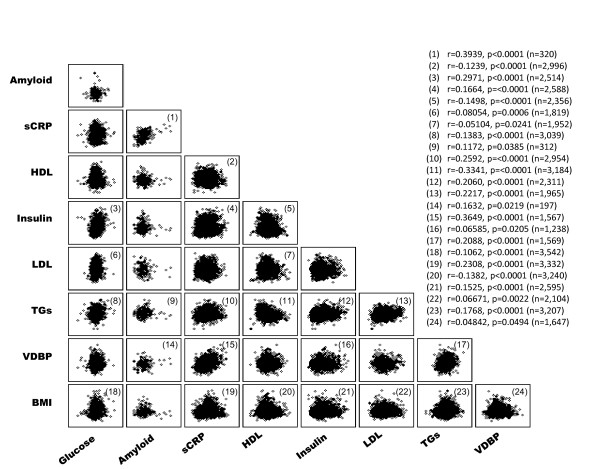
**Cross-correlation analysis between serum biochemical indices and metabolic risk factors of T2DM**. Only significant correlations are highlighted and numbered. For each significant correlation, Pearson's correlation coefficients (r), p-values and sample sizes are shown in parentheses.

In order to identify the association between a set of inflammatory markers and acute phase proteins (sCRP, amyloid-A, and VDBP) and the serum biochemical indices of predisposition to T2DM (fasting insulin and glucose, HDL-C, LDL-C, and TGs, as well as BMI), a cross-correlation analysis was carried out between these factors as shown in Figure 1. sCRP was significantly (*p < 0.0001*) correlated with increased levels of fasting insulin (*r *= 0.17), amyloid-A (*r *= 0.40), TGs (*r *= 0.26), and VDBP (*r *= 0.36) and with higher BMI (*r *= 0.23, *p < 0.0001*) but with lower levels of HDL-C (*r *= -0.12, *p < 0.0001*). As expected, circulating levels of HDL-C were inversely correlated with fasting insulin (*r *= -0.15, *p < 0.0001*), LDL-C (*r *= -0.05, *p = 0.024*) and TGs (*r *= *-0.33*, *p < 0.0001*). Furthermore, BMI was linked to elevated serum levels of fasting glucose (*r *= 0.11, *p < 0.0001*) and insulin (*r *= 0.15, *p < 0.0001*), LDL-C (*r *= 0.067, *p = 0.0022*), VDBP (r = 0.048, *p = 0.049*) and TGs (*r *= 0.18, *p < 0.0001*) and with lower serum HDL-C (*r *= -0.14, *p < 0.0001*).

## Discussion

We presented evidence for several associations between polymorphisms in various genes along the innate immunity-related inflammation cascade and the metabolic phenotypes associated with risk of T2DM. Several lines of evidence suggest a role for inflammation in regulating insulin action, glucose homeostasis and T2DM [7,21]. Compared to healthy individuals, subjects with T2DM risk factors such as obesity, hyper-triglyceridemia, or low HDL-C exhibit elevated serum levels of pro-inflammatory cytokines and acute-phase reactants [10]. Furthermore, it is well established that levels of inflammatory markers exhibit a gradual increase as T2DM develops and progresses to its complications. Initial high circulating levels of pro-inflammatory cytokines were also thought to contribute, at least partly, to the subsequent manifestation of disease [40-45].

Genetic variants in innate immunity-related inflammatory pathways may affect metabolic phenotypes associated with T2DM risk, and therefore contribute to disease pathogenesis. Indeed, a number of studies have noted that inherited variants in genes encoding for this pathway are associated with T2DM and other cardio-metabolic disorders [46-50]. We observed several associations between SNPs in genes along the innate immunity pathway and metabolic and phenotypic indices related to the risk of T2DM (Table 2). For example, fasting insulin levels were associated with SNPs in *IL6 *and *TNFA*, whereas HDL-C was associated with variants in *TNFA *and *CRP *(Table 2). The strongest associations observed in the present study were between two variants in *CRP *and serum levels of sCRP (*p < 0.0001*). The *CRP *variant rs1205 was inversely associated with sCRP, while rs1417938 exhibited a direct association with the serum levels of sCRP. *CRP *rs1417938 was also positively associated with circulating HDL-C and VDBP.

Previous reports have demonstrated a significant association between variants in the genes assessed here and serum levels of inflammatory markers, metabolic phenotypes associated with T2DM risk, and/or disease incidence (for review see 21). For example, genetic variants in the promoter region of the *IL6 *gene (rs1800795 and rs1800796; both are in LD with the *IL6 *SNPs examined here) have been linked with susceptibility to T2DM [26]. Furthermore, the association of polymorphisms in the *TNFA *with T2DM and its related complications has been extensively investigated [27-30]. The SNPs rs1800629 and rs361525, both located in the *TNFA *promoter region, have been implicated in the predisposition to T2DM [27]. The minor allele of rs1800629 has been linked to increased *TNFA *transcriptional activity [51], whereas rs361525 is located in a repressor site of the gene [52]. In this study, we noted a strong association between the *TNFA *variant rs3093662 and fasting insulin. Each additional allele of *TNFA *rs3093662 was found to be associated with about 37.1% (95%CI: 11.0-69.5%) increase in serum fasting insulin levels. Although *TNFA *rs3093662 was previously linked to a higher serum level of TNF-α [53], to our knowledge the present results is the first reported link between this variant and elevated levels of serum insulin. This observation substantiates a further investigation for the role of the *TNFA *gene variants in T2DM risk and progression.

A number of SNPs in the *CRP *gene have been linked to elevated levels of serum sCRP [32], insulin sensitivity [33], and T2DM [34,35]. As noted previously, in the present study we observed an association between two *CRP *variants and sCRP (rs1205 and rs1417938, Table 2). These variants markedly modulated the serum levels of sCRP where each additional allele of rs1205 conferred approximately 40% lowering in serum sCRP while an additional allele of rs1417938 resulted in about 67% increase in sCRP. These results are in line with observations indicating a strong impact of local SNPs of the *CRP *gene on plasma CRP levels although no direct evidence were established to demonstrate that these genetically controlled CRP elevations may contribute to cardiometabolic phenotypes [54]. However, several studies have reported that serum CRP, particularly in T2DM, can be influenced additively *via *the interaction between SNPs in *IL6 *and *CRP *[54-57]. Furthermore, a number of GWAS report have been carried out in order to identify loci that may modulate the serum levels of the metabolic indices such as LDL-C, HDL-C, CRP, TGs, etc. that were examined in this study [58-60]. Of the variants identified in these studies, only the *CRP *SNP rs2794520, which is in LD with rs1205, was examined in the present study and, as discussed earlier, was found to play a role in attenuating the serum levels of sCRP (Table 2).

Although several genetic variants were implicated in risk of T2DM, it is not clear whether these variants are themselves causal or in LD with other causal SNPs. A reason for not taking into account (Bonferroni correction) the number of phenotypes may be that the phenotypes are strongly correlated with each other. Further studies, such as pathway analyses, are required to understand the functional consequences of the present findings and to clarify the causal relationships between the studied (and other) variants along the innate-immunity-related inflammation and the risk of T2DM. This is essential with the present findings, particularly where several of the outcomes of association analysis (Table 2) were found to correlated with one another. In addition, identification of additional SNPs or copy number variations (CNVs) of inflammation-related genes, their frequencies and their association with risk of T2DM will assist in further clarifying the genetic basis of the disease and in designing strategies for early risk prediction. Gene-gene interactions may present a potential to partly explain phenotypic variability and disease risk. Although we only investigated one such instance in this analysis (*i.e*., the interaction between *CRP *polymorphisms affecting circulating sCRP levels), with improved methodologies for large scale gene-gene interaction analysis, additional analyses may prove valuable in elucidating mechanisms by which T2DM develops, as well as in improving risk prediction [61]. Gene-environment interactions, and in particular gene-diet interactions, may also contribute to inter-individual variability in disease risk, and the potential modifying effects of nutrients with anti-inflammatory properties, such as vitamin D, on the relationship between genetic variants and metabolic phenotypes associated with increased T2DM risk are being currently investigated. A potential limitation of this study stems from its focus on a selected panel of SNPs without investigating a more comprehensive set of genome-wide variants in association with the metabolic trait of interest. This candidate gene approach is known to have a limited power in identifying novel loci compared to a genome-wide association scan (GWAS). Moreover, investigating only a few SNPs along a particular pathway (e.g., innate immunity-related inflammation as investigated here) will preclude the assessment of interactions between multiple related pathways on disease etiology (e.g., various metabolic and phenotypic indices related to risk of T2DM). Our choice, however, of SNPs along the inflammatory cascade was substantiated from numerous reports implicating this signaling pathway in susceptibility to T2DM [22,26-35].

The results of the present study demonstrate inter-correlations between various metabolic phenotypes that are linked to T2DM risk. These correlations include associations between biomarkers of inflammation such as sCRP (whose serum levels increase as a downstream effect of the action of pro-inflammatory cytokines) and dysregulation of lipid metabolism, such as increased fasting glucose and insulin, decreased HDL-C and increased serum TGs (Figure 1). The observed correlations between the assessed metabolic phenotypes of T2DM risk are in agreement with existing knowledge with respect to the clinical interaction between these biomarkers [62,63]. Furthermore, the inter-correlation between the different metabolic and phenotypic indices of disease risk provides an integrated view of the T2DM risk profile as being determined by the dysregulation of several physiologic pathways, such as glucose metabolism, lipid metabolism, and the inflammatory response. This view is also supported by our observation that several of the SNPs assessed here were associated with phenotypes from multiple metabolic pathways. In this respect, we observed a significant correlation between increased BMI and biochemical indices of T2DM (*e.g.*, measures of glucose, lipid metabolism, and inflammatory markers; see Figure 1). This result is agreement with previous research implicating obesity in the predisposition to T2DM [7,44] and suggest the use of BMI as a robust surrogate of cardiometabolic dysfunction. Extensive experimental, clinical and epidemiological studies have previously linked obesity to the activation of inflammatory signalling pathways and to the subsequent manifestations of T2DM [7,64,65]. This observation further suggests a possible role of the innate immunity-related inflammatory pathway in the etiology of T2DM and implicates factors that attenuate inflammation as an approach for disease intervention [21].

## Conclusions

In conclusion, our results support previous evidence demonstrating that genetic variants along innate immune-related inflammatory pathways are associated with metabolic risk factor phenotypes for T2DM. This research contributes toward the knowledge base for biomarker discovery for early T2DM detection. The findings from this study also highlight multiple associations between biochemical indices of T2DM and suggest an underlying network of various pathways that become dysregulated during the onset and progression of the disease.

## Competing interests

The authors declare that they have no competing interests.

## Authors' contributions

PA, BGB and ZD participated in study design, performed data analysis and interpretation, and drafted the manuscript. DB performed data analysis and interpretation. AV selected the proxies of the candidate SNPs in the genes of interest. SM contributed in developing the collaboration between PHAC and McGill University. TDS supervised cohort recruitment and genotyping. MK, BR and AE-S participated in study design and data interpretation and coordination. AB conceived of the study, participated in its design and coordination and in data interpretation and manuscript writing. All authors provided critical manuscript revisions, read and approved the final manuscript.

## Pre-publication history

The pre-publication history for this paper can be accessed here:

http://www.biomedcentral.com/1471-2350/12/95/prepub

## Supplementary Material

Additional file 1 **Figure S1**. LD structure for all genotyped SNPs from hapmap CEU population in the studied genes http://www.hapmap.org. (a) *TNFA*, (b) *CRP*, (c) *IL1A*, (d) *IL6*, (e) *TLR4* Click here for file

Additional file 2 **Table S1**. Frequencies and means of metabolic markers across genotypes. Click here for file
