# Supplementary figures and images for: Genetic polymorphisms of innate immunity-related inflammatory pathways and their association with factors related to type 2 diabetes
Source: BMC Med Genet. 2011 Jul 14;12:95. doi: 10.1186/1471-2350-12-95 (PMC3161932; doi:10.1186/1471-2350-12-95)

**Web Figure 1**

a)


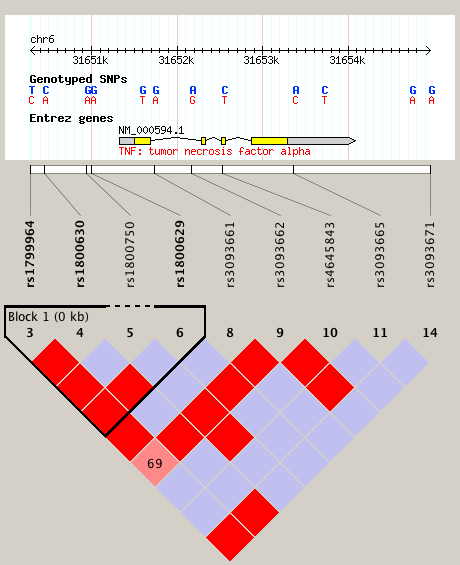


b)


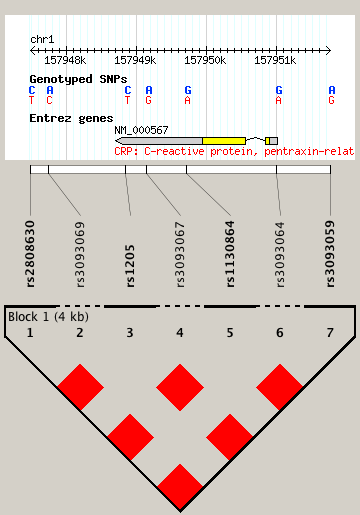


**Web Figure 1 cont’d**

c)


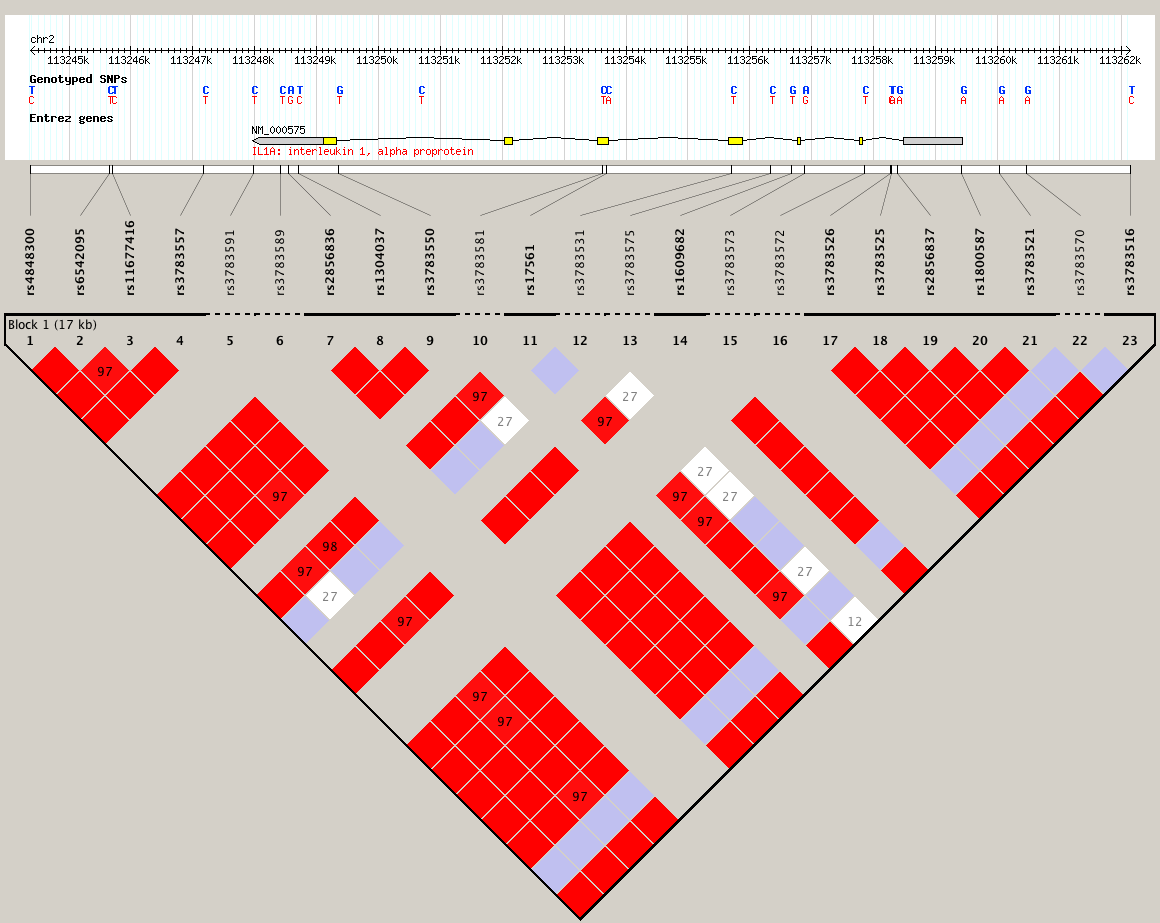


d)


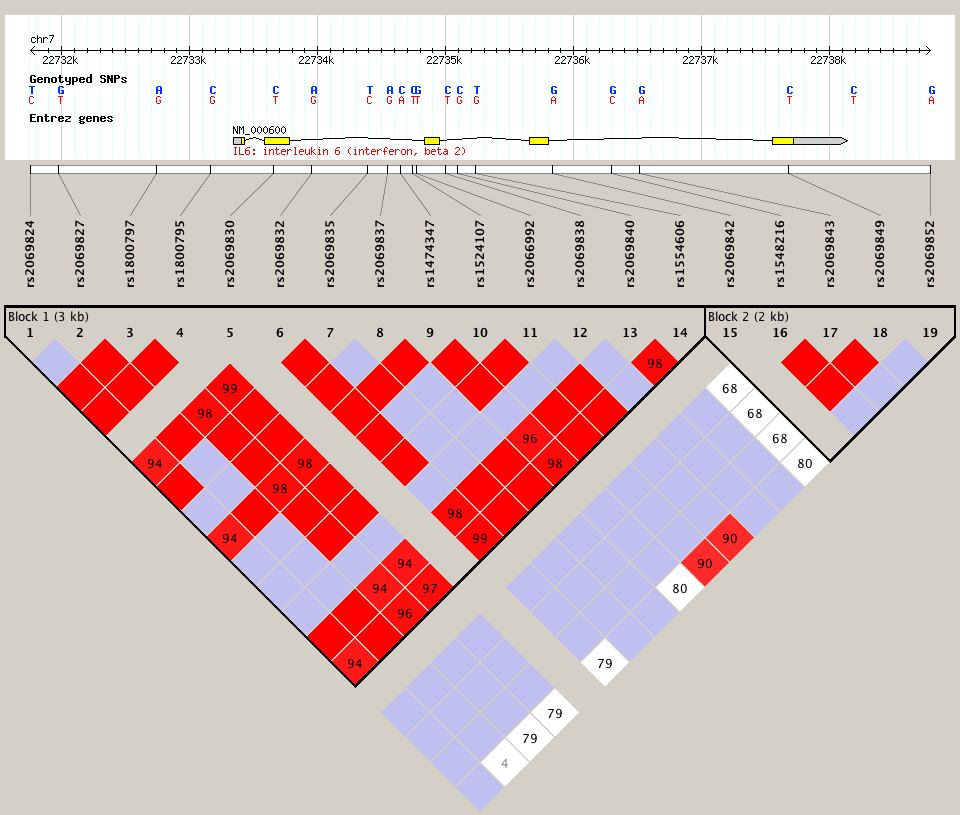


**Web Figure 1 cont’d**

e)


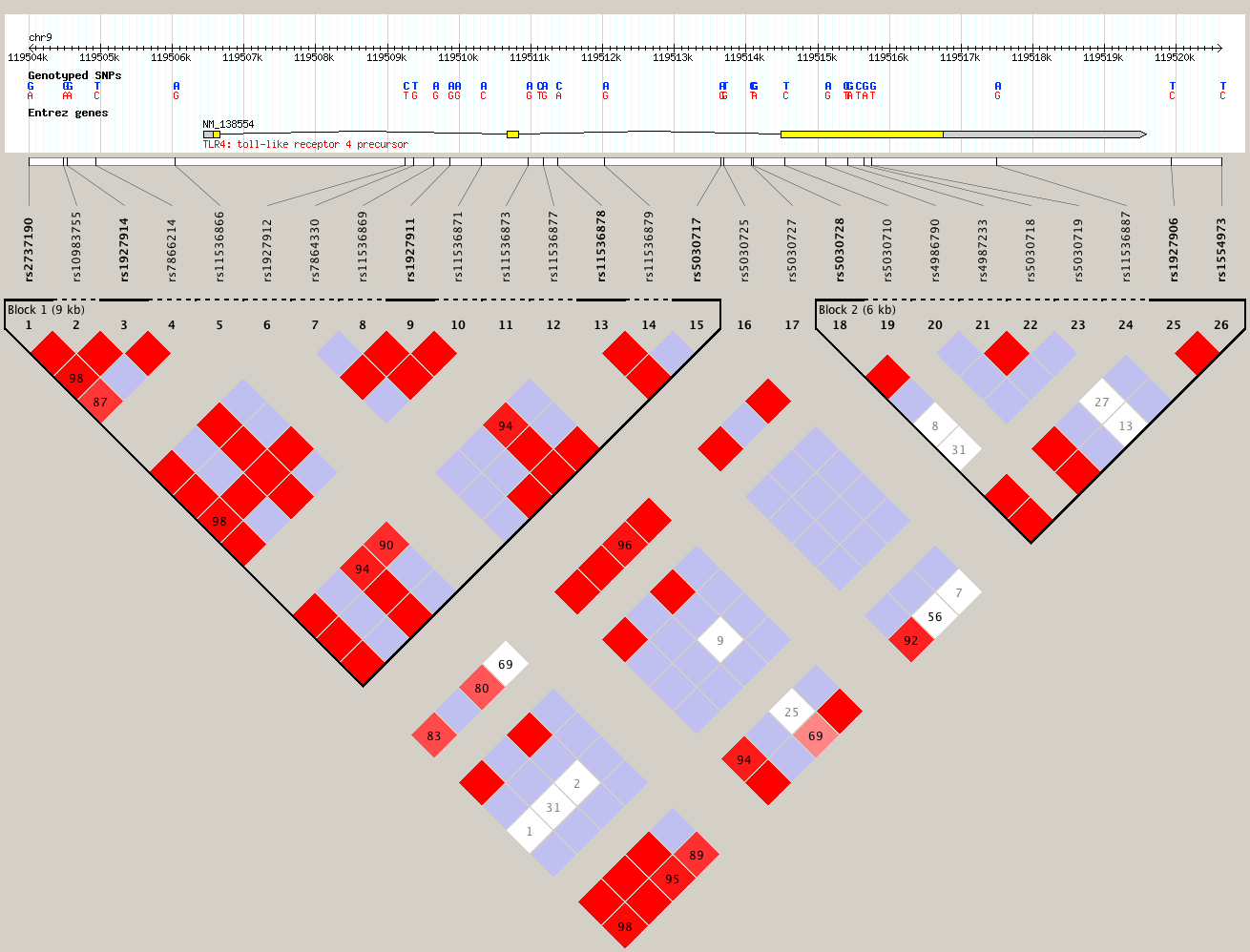

Supplement: Additional file 1 — Figure S1. LD structure for all genotyped SNPs from hapmap CEU population in the studied genes http://www.hapmap.org. (a) TNFA, (b) CRP, (c) IL1A, (d) IL6, (e) TLR4 [file 1471-2350-12-95-S1.DOC]
